# Novel heavily fucosylated glycans as a promising therapeutic target in colorectal cancer

**DOI:** 10.1186/s12967-023-04363-5

**Published:** 2023-07-26

**Authors:** Kuei-Yen Tsai, Yu-Jia Chang, Chien-Yu Huang, G. M. Shazzad Hossain Prince, Hsin-An Chen, Precious Takondwa Makondi, Ying-Rou Shen, Po-Li Wei

**Affiliations:** 1 Graduate Institute of Clinical Medicine, College of Medicine, Taipei Medical University, Taipei, 11031 Taiwan; 2 Department of Surgery, School of Medicine, College of Medicine, Taipei Medical University, Taipei, 11031 Taiwan; 3 Division of General Surgery, Department of Surgery, Shuang Ho Hospital, Taipei Medical University, New Taipei City, 235041 Taiwan; 4 Cancer Research Center and Translational Laboratory, Department of Medical Research, Taipei Medical University Hospital, Taipei Medical University, Taipei, 11031 Taiwan; 5 Department of Pathology, Wan Fang Hospital, Taipei Medical University, Taipei, 11696 Taiwan; 6 Cell Physiology and Molecular Image Research Center, Wan Fang Hospital, Taipei Medical University, Taipei, 116 Taiwan; 7 School of Medicine, National Tsing Hua University, Hsinchu, 30013 Taiwan; 8 Institute of Molecular and Cellular Biology, National Tsing Hua University, Hsinchu, 30013 Taiwan; 9 Kamuzu Central Hospital, National Cancer Center, P.O. Box 149, Lilongwe, Malawi; 10 Research Department, GlycoNex Inc., New Taipei City, 22175 Taiwan; 11 Division of Colorectal Surgery, Department of Surgery, Taipei Medical University Hospital, Taipei Medical University, 252 Wuxing Street, Sinyi District, Taipei, 11031 Taiwan; 12 Graduate Institute of Cancer Biology and Drug Discovery, Taipei Medical University, Taipei, 11031 Taiwan

**Keywords:** CRC, Anti-HFG mAb, Fucosylated glycans

## Abstract

**Background:**

Colorectal cancer (CRC) is highly prevalent and lethal globally, and its prognosis remains unsatisfactory. Drug resistance is regarded as the main cause of treatment failure leading to tumor recurrence and metastasis. The overexpression of fucosylated epitopes, which are usually modifications of glycoproteins, was reported to occur in various epithelial cancers. However, the effects of treatments that target these antigens in colorectal cancer remain unclear.

**Methods:**

This study investigated the expression of heavily fucosylated glycans (HFGs) in 30 clinical samples from patients with CRC and other normal human tissues. The complement-dependent cytotoxicity was explored in vitro through treatment with anti-HFG monoclonal antibody (mAb) alone or in combination with chemotherapeutic agents. In vivo inhibitory effects were also examined using a xenograft mouse model.

**Results:**

Immunohistochemistry staining and western blotting revealed that HFG expression was higher in human colorectal cancer tissues than in normal tissues. In DLD-1 and SW1116 cells, which overexpress fucosylated epitopes, anti-HFG mAb produced observable cytotoxic effects, especially when it was combined with chemotherapeutic agents. The xenograft model also demonstrated that anti-HFG mAb had potent and dose-dependent inhibitory effects on colorectal tumor growth.

**Conclusions:**

As a novel cancer antigen, HFGs are a promising treatment target, and the implementation of anti-HFG mAb treatment for CRC warrants further investigation.

**Supplementary Information:**

The online version contains supplementary material available at 10.1186/s12967-023-04363-5.

## Introduction

In 2020, approximately 1.9 million newly diagnosed colorectal cancer (CRC) cases and more than 900 000 deaths from CRC were reported worldwide, accounting for 10% of cancer incidence and 9.4% of total cancer-related deaths in that year [1]. Clinically, surgery remains the standard treatment for CRC, while radiotherapy, systemic medical treatment, or a combination of both may also be administered according to tumor location and stage [2]. For metastatic CRC (mCRC), oxaliplatin- and irinotecan-based regimens are frequently used as first-line chemotherapy. However, drug resistance and intolerance of the adverse effects (e.g., hair loss, diarrhea, neurotoxicity, and hand-foot syndrome) have been reported [3]. Furthermore, drug resistance is the leading cause of cancer relapse and disease progression, and biological agents have been developed to improve therapeutic responses [4, 5]. Targeted agents against vascular endothelial growth factor (VEGF) or epidermal growth factor receptor (EGFR) are used in combination with cytotoxic therapy as a first-line treatment for mCRC [6]. However, only a few target agents are applied in clinical, and the overall survival rate of patients with distant metastasis is only 14% [7]. Therefore, developing a new drug for treating CRC is a crucial task.

Studies have reported that increased sialylation, galactosylation, and fucosylation are correlated with CRC progression [8]. Tumor-associated fucosylated epitopes are a class of carbohydrate molecules and include Lewis a, Lewis y, Lewis x, and sialyl Lewis antigens [9]. Among various features, fucosylation is among the most frequently occurring modifications in glycoproteins and glycolipids, and overexpression of fucosylated epitopes, including certain Lewis antigens, was detected in various epithelial cancers [10]. The activity of fucosyltransferase (FUT) 3 and 6 promotes transforming-growth-factor-ß-mediated CRC cells through the epithelial–mesenchymal transition (EMT) and metastatic tendency, and the tumors in patients with advanced clinical stage CRC and vascular invasion exhibit high levels of fucosylated proteins [11]. The expression of Lewis y was also detected in CRC tumors and revealed to be strongest in stage IV tumors [12]. Among patients with CRC, nonresponders to irinotecan/5-fluorouracil (5-FU)/leucovorin exhibited considerably higher expression of sialyl Lewis^X^ [13]. Therefore, the overexpression of fucosylated epitopes is associated with tumor invasion, metastasis, poor prognosis, and drug resistance [14, 15].

In the present study, a humanized monoclonal antibody (mAb) was designed that specifically binds to novel heavily fucosylated glycans (HFGs). This novel fucosylated epitope, HFG, is a Lewis antigen–related glycan that is characterized by the presence of multiple fucose residues. Immunohistochemistry (IHC) and western blotting were performed to assess the expression level of HFG in tumor tissues and in normal tissues adjacent to tumors (NATs). Furthermore, we assessed the feasibility of chemotherapy drugs combined with anti-HFG mAb in CRC cells and demonstrated that this novel HFG is a potential tumor marker of CRC and a promising therapeutic target.

## Materials and methods

### Human samples

The present study was approved by the Institutional Review Board of Taipei Medical University Hospital (N201906007). Thirty patients with CRC and 10 healthy donors were randomly selected and recruited for the present study; their informed consent was obtained. Tumor tissues and NATs were collected between July 2019 and February 2020 during the surgical resection of the 30 patients with CRC at Taipei Medical University Hospital. Each NAT was sampled at least 10 cm from the tumor margin, and NATs were not allowed to exhibit the characteristics of malignant histopathology. All the tissue specimens were fixed with 10% formalin and embedded in paraffin blocks. Blood samples were collected from the 10 healthy donors and 30 patients with CRC before surgery and approximately 1 month after surgery between July 2019 and March 2020. Plasma samples were harvested by centrifuging the blood specimens at 1710 × *g* for 15 min at ambient temperature; the samples were then stored at − 80 °C until use.

### IHC and scoring criteria

The tissue blocks from CRC patients were cut into 4-μm-thick sections and affixed onto slides. The tissue sections were deparaffinized with xylene, rehydrated using gradient alcohol, and then boiled in antigen retrieval buffer (10 mM citric acid and 0.05% Tween-20, pH 6.0) for 30 min. After three washes with phosphate-buffered saline (PBS), the sections were immersed in PBS containing 3% hydrogen peroxide (Sigma) to quench endogenous peroxidase activity.

Anti-HFG mAb developed by GlycoNex Inc. was used to detect the expression of HFG. For the IHC analysis, the tissue slides of CRC patients and the human normal tissue microarray FDA999 (US Biomax) were incubated at 4 °C overnight with the primary antibody anti-HFG mAb at 20 and 2 μg/mL, respectively. Subsequently, after three washes with PBS, the slides were incubated for an hour with a secondary mouse anti-human IgG Fc-HRP antibody (Southern Biotech), at a dilution of 1:2,000 and then visualized using a DAB-Plus Substrate Kit (Dako). The scoring of patients’ tissues included the intensity and percentage of positively stained cells. Staining intensity was denoted as 0 (negative), 1 (weakly positive), 2 (moderately positive), or 3 (strongly positive). Staining percentage was examined based on the estimated percentage of stained tumor cells among the total tumor cells or the estimated percentage of stained epithelial cells among the total epithelial cells. As for the scoring of FDA999, the average staining intensity was presented from + to +  +  + , while – indicated negative staining. The number of positive stains in all tissues were also present in brackets.

Human multiple cancer tissue microarrays BC000120, BC001128, and ST2091 were purchased from US Biomax. The tissue sections were first deparaffinized with xylene, rehydrated through gradient alcohol, and then boiled in antigen retrieval buffer (10 mM citric acid, 0.1% NP-40, pH 6.0) for 30 min. After washing three times with PBS, the sections were immersed in PBS containing 3% hydrogen peroxide (Sigma) to quench endogenous peroxidase activity. For IHC analysis, the slides were incubated with a primary antibody anti-HFG mAb (10 μg/mL) at room temperature for an hour. Then, after washing three times with PBS, the slides were incubated with a secondary mouse anti-human IgG (Fc)-BIOT antibody (Southern Biotech), at a dilution of 1:2,000 for an hour followed by VECTASTAIN® Elite® ABC HRP Kit (Vector Laboratories) and then visualized by DAB-Plus Substrate Kit (Dako). The staining was scored and further calculated as positive rates of staining because the tissue numbers of different cancer types varied.

### HFG expression on human cancer cell lines by flow cytometry

Human cancer cell lines were obtained from the American Type Culture Collection (ATCC), Bioresource Collection and Research Center (BCRC) of the Food Industry Research and Development Institute (FIRDI, Hsinchu, Taiwan), and Japanese Collection of Research Bio-resources Cell Bank (JCRB) and then cultured according to standard mammalian tissue culture protocols and sterile technique. Each human cancer cell line was suspended in PBS with 2% heat-inactivated FBS (Hyclone) and added to a V-shape 96-well plate (Nunc), followed by the addition of equal volume of anti-HFG mAb. The plates were then incubated at 4 °C for an hour. After washing with PBS once, the plates were centrifuged, and the cell pellet was re-suspended in 200-fold PBS-diluted Fluorescein (FITC)-AffiniPure Goat Anti-Human IgG, Fcγ Fragment Specific (Jackson ImmunoResearch Inc) and incubated at 4 °C for 30 min. Then, the plates were centrifuged, and PBS was added to the wells to wash off unbound secondary antibodies. The cell pellet was re-suspended in ice-cold PBS and then analyzed on a FACS Canto cytometer instrument and FACS Diva software (BD Biosciences).

### Protein extraction and Western blot analysis

Tissues were cut into pieces with sterilized scissors and washed three times with saline. They were then lysed using lysis buffer (1% Triton X-100 in 50-mM Tris–HCl and 0.15-M NaCl, pH 7.5), homogenized with TissueRuptor (QIAGEN), and agitated at 4 °C for 1 h. AGS and MKN45 cells were scraped from cell culture flasks and then washed three times with saline. Cells were lysed using lysis buffer and agitated at 4 °C for 30 min. Tissue and cell lysates were centrifuged at 15,521 × *g* and 4 °C for 15 min to remove debris. The supernatant was collected, and the concentration of lysates was determined using Pierce 660 nm Protein Assay Reagent (Thermo Scientific), MOPS running buffer (M00138, Genescript), ExpressPlus PAGE Gel (M42015, Genescript), and SurePAGE (M00657, Genescript) in sodium dodecyl sulfate–polyacrylamide gel electrophoresis. Tissue lysates (10 μg) or cell lysates (20 μg) were mixed with 1 × NuPAGE LDS sample buffer (NP0007, Invitrogen) and 1 × sample reducing agent (NP0009, Invitrogen). The protein samples were boiled at 100 °C for 10 min before being loaded into 4–20% gel, run at 80 V for 20 min, and then run at 110 V for 90 min. Proteins were transferred to polyvinylidene difluoride membrane (BSP0161, PALL) at 150 mA for 180 min. Immunoblotting was performed by conducting blocking with 5% skim milk at room temperature for 1 h, incubation overnight with 10 μg/mL of primary antibody anti-HFG mAb at 4 °C, and incubation with the secondary antibody mouse anti-human IgG Fc-HRP (1:5000, Southern Biotech) at ambient temperature for 1 h. Subsequently, the membranes were washed four times for 5 min with wash buffer (0.05% Tween-20 in PBS). Signals were visualized with electrochemiluminescence reagent (NEL105001EA, PerkinElmer).

### Cell culture and treatment

DLD-1, SW1116, COLO 201, and COLO 205 cell lines were purchased from the American Type Culture Collection. The DLD-1 cells were maintained in complete RPMI-1640 medium (Gibco) supplemented with 10% heat-inactivated fetal bovine serum (FBS, Hyclone) and cultured at 37 °C in a humidified incubator with 5% CO_2_. The SW1116 cells were maintained in Leibovitz's L-15 medium (Gibco) supplemented with 10% heat-inactivated FBS and cultured at 37 °C in a humidified incubator. All cell lines included in the study was summarized in Additional file 1: Table S1 with individual mutational status. The chemotherapy drugs used in the present study included 5-FU (Nang Kuang Pharmaceutical), oxaliplatin (Oxalip, TTY Biopharm), and irinotecan (Campto, Pfizer). DLD-1 and SW1116 cells were seeded at 5 × 10^3^ and 2 × 10^4^ cells/well, respectively, in 96-well culture plates (Greiner Bio-One). After they were incubated overnight, the cells were treated with either vehicle, a combination of 5-FU plus oxaliplatin, or a combination of 5-FU plus irinotecan and then incubated in a 37 °C humidified incubator for 48 h. All drugs were diluted in the aforementioned cell culture medium. Chemotherapy drug treatments were divided into low-concentration and high-concentration groups. Among the cells treated with low concentrations of chemotherapy drugs, the DLD-1 cells were treated with 1 μM 5-FU and 1.5 μM oxaliplatin or 1 μM 5-FU and 20 μM irinotecan, whereas the SW1116 cells were treated with 5 μM 5-FU and 30 μM oxaliplatin or 5 μM 5-FU and 60 μM irinotecan. Among the cells treated with high concentrations of chemotherapy drugs, the DLD-1 cells were treated with 5 μM 5-FU and 6 μM oxaliplatin or 5 μM 5-FU and 60 μM irinotecan, whereas the SW1116 cells were treated with 75 μM 5-FU and 50 μM oxaliplatin or 75 μM 5-FU and 100 μM irinotecan.

### Complement-dependent cytotoxicity (CDC) assay

After the cells were incubated with chemotherapy drugs for 48 h, their supernatant was discarded. Subsequently, 50 μL of the medium, 50 μL of anti-HFG mAb (at the final concentration of 25, 100, or 400 μg/mL), and 50 μL of normal human serum complement (QUIDEL) were added to a 96-well plate sequentially. The plate was incubated at 37 °C for 4 h. The cytolysis of DLD-1 and SW1116 cells was analyzed using the CellTiter-Glo Luminescent Cell Viability Assay (Promega) in accordance with the manufacturer’s instructions. The cell viability of control well–indicated DLD-1 and SW1116 cells not treated with chemotherapy drugs or anti-HFG mAb-induced CDC was evaluated using the following formula: cell viability (%) = (experimental well/control well) × 100%.

### In vivo CRC xenograft model

Animal experiments were performed strictly in accordance with the regulations of the Institutional Animal Care and Use Committee (IACUC) of GlycoNex. Specific-pathogen-free female CB17 severe-combined-immunodeficiency (SCID) mice were purchased from BioLASCO (Taiwan). DLD-1, COLO 201, and COLO 205 cells were resuspended at a cell density of 5 × 10^6^ cells per 200 μL of ice-cold serum-free medium. All the mice were injected subcutaneously in the flank region with 200 μL of cell suspension when they were aged 6–8 weeks. Their body weight was measured once weekly, their tumor size was measured weekly with a digital caliper, and their tumor weight was estimated using the following formula: weight in mg = (width^2^ × length) mm^3^/2. Because of ethical considerations pertaining to animal experiments, a tumor burden of > 10% of body weight and a body weight loss of > 15% were defined as the humane endpoints.

When the tumor weight of the tumor-bearing SCID mice reached 150–200 mg, they were intraperitoneally injected with anti-HFG mAb or saline, which served as a negative control. In the DLD-1 xenograft model, mice (n = 4) were intraperitoneally injected with 50 mg/kg of anti-HFG mAb twice weekly for 5 weeks. In the COLO 205 xenograft model, mice (n = 6) were intraperitoneally injected with 50 mg/kg of anti-HFG mAb twice weekly for 5 weeks. In the COLO 201 xenograft models, mice (n = 6) were intraperitoneally injected with 2, 10, and 50 mg/kg of anti-HFG mAb twice weekly for 6 weeks or 0.008, 0.04, 0.2, and 1 mg/kg of anti-HFG mAb once weekly for 6 weeks. In all the xenograft models, the first dose of anti-HFG mAb was 1.5 times the indicated dose. Antibodies were diluted with saline (20 μL per gram of body weight was intraperitoneally injected).

### Statistics

The collected data were analyzed using the statistics functions of Microsoft Excel. Values are presented as the mean ± standard deviation (SD). Continuous variable data were analyzed with one-way analyses of variance or two-tailed independent t-tests to perform comparisons of two groups. A *p*-value of < 0.05 was regarded to indicate a statistically significant difference (* = *p* < 0.05; ** = *p* < 0.01).

## Results

### Higher levels of HFGs exist in tumor tissues of patients with CRC

**Fig. 1:**
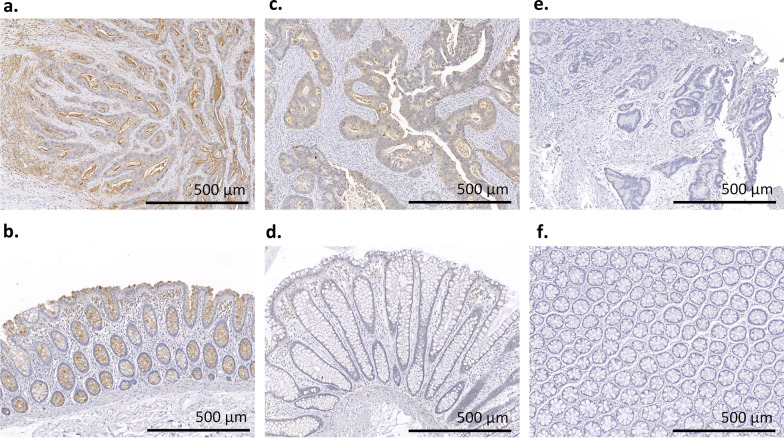
IHC staining of HFGs by anti-HFG mAb in NATs and tumor tissues of three patients with CRC. In patient-19, both the tumor tissue **a** and NAT **b** are positively stained. In patient 5, the tumor tissue **c** and NAT **d** are positively and negatively stained, respectively. In patient-21, both the tumor tissue (e) and NAT **f** are negatively stained. *CRC* colorectal cancer, *HFG* heavily fucosylated glycan, *IHC* immunohistochemistry, *NAT* normal tissue adjacent to tumor

**Fig. 2:**
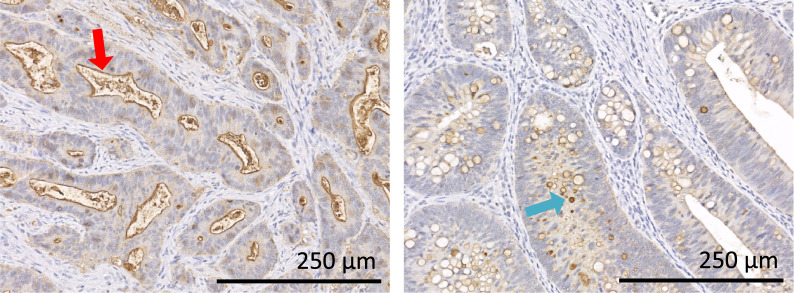
IHC staining of HFGs by anti-HFG mAb in tumor tissues of two patients with CRC. Positive signals are observed mainly in the cell membrane (a, red arrow, patient-19) and in cytoplasm (b, blue arrow, patient-5) of tumor cells. *CRC* colorectal cancer, *HFG* heavily fucosylated glycan, *IHC* immunohistochemistry

To identify the expression of HFG in NATs and CRC tumor tissues, the tissue sections from 30 patients with CRC were stained with anti-HFG mAb (i.e., a mAb for recognizing HFGs) through IHC. Table 1 lists the scores for each patient’s NAT and tumor tissue. The clinical and pathological characteristics were summarized in Additional file 1: Table S2. A score could not be obtained for the tumor tissue of patient-1 because of a lack of tumor cells in the tumor tissue. High levels of HFGs were expressed in all patients except patient-11 and patient-24, in whom HFG expression was higher in their NAT than in their CRC tumor tissue. Furthermore, the IHC staining results for patient-19, patient-5, and patient-21, presented in Fig. 1, highlight three conditions, namely positive HFG staining in NAT and tumor tissue (Fig. 1a, b), negative HFG staining in NAT and positive HFG staining in tumor tissue (Fig. 1c, d), and negative HFG staining in both tumor tissue and NAT (Fig. 1e, f). For patient-19 and patient-5, HFGs were revealed to be mainly localized in the cell membrane and in the cytoplasm of tumor cells (Fig. 2). Among the patients, 90% (26/29) of the tumor tissues from 29 patients expressed HFGs and that only 30% (9/30) of these patients’ NATs expressed HFGs. The IHC results for paired NAT and tumor tissue are listed in Table 2; they indicate that positive staining of tumor tissue and negative staining of NAT were discovered in 59% of the patients, positive staining of both tumor tissue and NAT in 31%, and negative staining of both tumor tissue and NAT in 10%. No negative staining of tumor tissue and positive staining of NAT were found in any of the patients' samples.

**Table 1:** IHC detection of HFGs by anti-HFG mAb in NATs and tumor tissues of patients with CRC

Tissue	NAT		Tumor	
Patient no.	Score	%	Score	%
1	0	–	N/A	N/A
2	0	–	1	40
3	0	–	2	100
4	0	–	2	80
5	0	–	2	80
6	0	–	1	30
7	0	–	2	30
8	3	80	3	100
9	0	–	1	60
10	1	10	2	60
11	3	95	2	15
12	2	70	3	100
13	0	–	0	–
14	0	–	2	100
15	1	15	1	100
16	3	60	2	100
17	0	–	1	60
18	0	–	2	90
19	3	70	3	100
20	0	–	1	10
21	0	–	0	–
22	0	–	1	20
23	0	–	1	80
24	3	100	2	75
25	3	30	2	100
26	0	–	1	40
27	0	–	2	100
28	0	–	0	–
29	0	–	2	80
30	0	–	1	20

*CRC* colorectal cancer, *HFG* heavily fucosylated glycan, *IHC* immunohistochemistry, *NAT* normal tissue adjacent to tumor, *N/A* not available due to lack of tumor cells in tumor tissue

**Table 2:** IHC detection of HFGs by anti-HFG mAb in paired NAT and tumor tissues of 29 patients with CRC

Staining of tumor tissue versus NAT (n = 29)		Tumor tissue	
		Positive (%)	Negative
NAT	Positive	9 (31)	0
	Negative	17 (59)	3 (10%)

*CRC* colorectal cancer, *HFG* heavily fucosylated glycan, *IHC* immunohistochemistry, *NAT* normal tissue adjacent to tumor

**Fig. 3:**
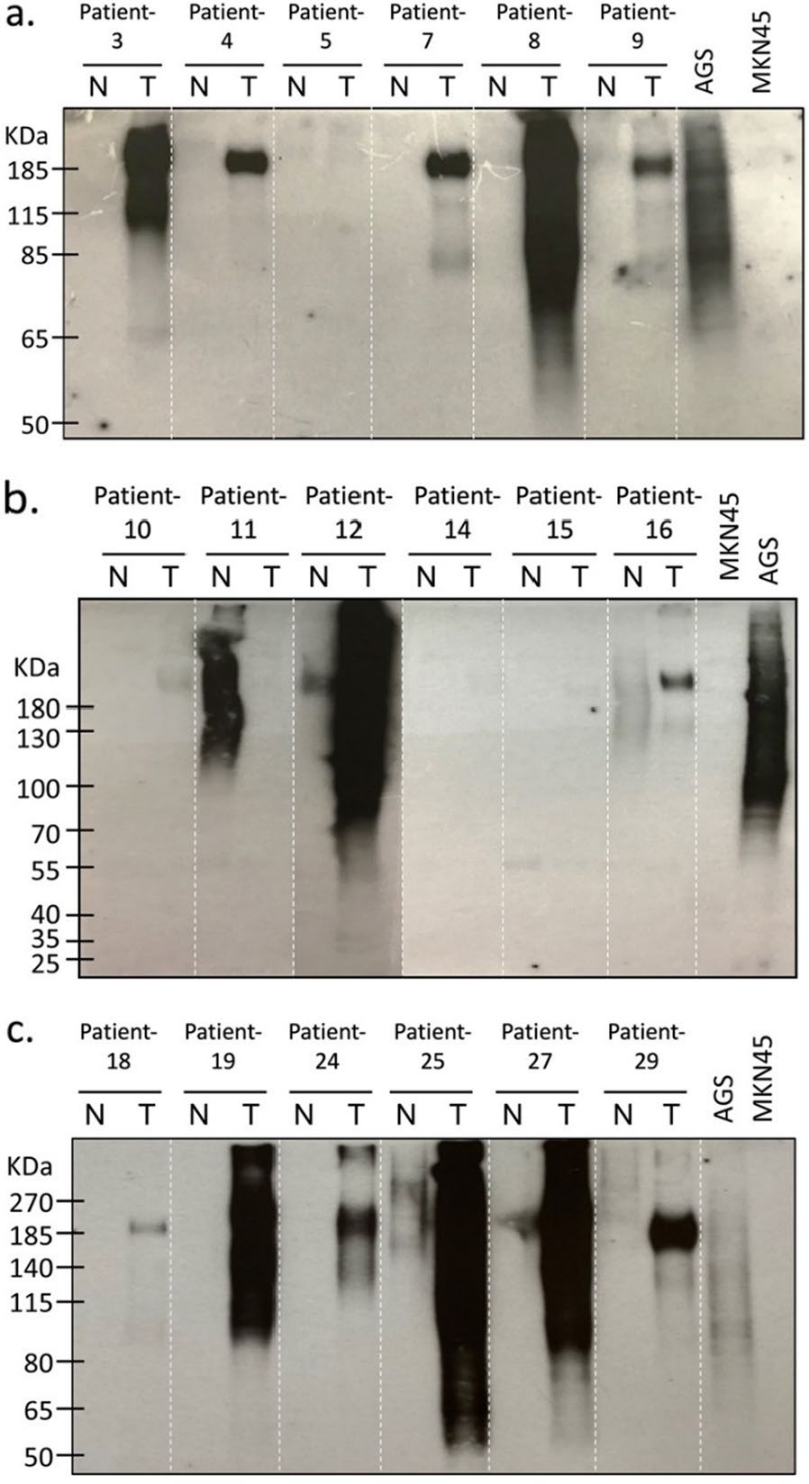
**a**–**c** Western blot analysis of HFGs by anti-HFG mAb in NAT and tumor tissues of 18 patients with CRC. *CRC* colorectal cancer, *HFG* heavily fucosylated glycan, *IHC* immunohistochemistry, *N* normal tissue adjacent to tumor, *T* tumor tissue; AGS, cells with high expression of HFGs that can be recognized by anti-HFG mAb (positive control); MKN45, cells without the expression of HFGs (negative control)

The IHC results revealed that HFGs were detected in 90% of CRC tumor tissues; the HFG expression of 18 patients was further verified through western blotting. Tissue lysates from the 18 selected patients were analyzed with anti-HFG mAb; Fig. 3 presents the adjoined results (for NAT and tumor tissue) for each selected patient. The western blot results indicated higher levels of heavily fucosylated glycoproteins in tumor tissue than in NAT for 13 patients. Furthermore, a strong major band near 185 kDa was observed in the tumor tissue of seven patients (patient-4, patient 7, patient-9, patient-16, patient-18, patient-24, and patient-29). By contrast, a stronger HFG signal was detected in the NAT of patient-11, which was compatible with the patient’s IHC staining score. For patient-5, patient-14, and patient-15, neither their NAT nor their tumor tissue produced signals of heavily fucosylated glycoproteins. The western blot and IHC results were mostly consistent except for those pertaining to the tumor tissue of patient-5 (IHC score, 2; Western blot, negative) and the NATs of patient-8, patient-19, and patient-24 (IHC score, 3; Western blot, negative). These differences could have been caused by the heterogeneous expression of HFGs in tissues. The use of different tissue parts for the two methods could have led to different expression levels. On the basis of the IHC and western blot analysis results, HFG was recognized by the anti-HFG mAb to be overexpressed in the tumor tissue of patients with CRC in comparison to that in their NAT.

### Overexpression of fucosylated antigens in human CRC cells instead of normal colonic epithelial cells

A study indicated that Lewis antigens are expressed at moderate levels in healthy human tissues (e.g., reproductive and digestive epithelial cells) but overexpressed on the cell surface of various cancers (e.g., lymphoma or malignant neoplasm of the breast, lung, liver, pancreas, kidney, bladder, or prostate) [10]. To determine the fucosylated antigen levels in normal and cancerous tissue, the anti-HFG mAb was used to perform immunostaining of both healthy and cancerous samples. The staining results revealed higher levels of fucosylated antigen in the salivary glands, larynx, esophagus, stomach, pancreas, and small intestine. However, the results indicated low levels of fucosylated antigens in normal colon tissue (Table 3). By contrast, strong anti-HFG mAb signaling was produced by CRC tissues, especially in adenocarcinoma of the colon (Table 4). These findings are consistent with our IHC staining results for HFGs. In subsequent experiments, several adenocarcinoma CRC cell lines—COLO 205, COLO 201, SW1116, DLD-1, LS 174 T, and HT-29—were evaluated through anti-HFG mAb epitope expression (Table 5). Strong binding affinity was detected in SW1116 and DLD-1 cells, whereas no binding affinity was detected in HT-29 or COLO 205 cells. Collectively, fucosylated antigens were overexpressed in human CRC cells rather than normal colonic epithelial cells.

**Table 3:** Anti-HFG mAb immunostaining of various human normal tissues

Tissue	anti-HFG mAb
Pancreas	± (3/3)
Tongue (Salivary gland tissue)	+ (3/3)
Larynx	+ + (3/3)
Esophagus	± (3/3)
Stomach	+ (3/3)
Small intestine	+ + + (3/3)
Colon	−
Hypophysis	−
Breast	−
Cerebrum	−
Cerebellum	−
Adrenal gland	−
Parathyroid gland	−
Ovary	−
Testis	−
Spleen	−
Tonsil	−
Thymus gland	−
Bone marrow	−
Lung	−
Heart	−
Liver	−
Kidney	−
Prostate	−
Uterus	−
Uterine cervix	−
Striated muscle	−
Skin	−
Nerve	−
Greater omentum	−
Endometrium	−

**Table 4:** Anti-HFG mAb immunostaining of human cancerous tissues

Organ	Pathology diagnosis	Type	Positive rate		
Stomach	Adenocarcinoma	Malignant	57/175	32.6%	33.2%
	Mucinous adenocarcinoma		6/17	35.3%	
	Undifferentiated adenocarcinoma		1/2	50.0%	
	Signet-ring cell carcinoma		1/2	50.0%	
Colon	Adenocarcinoma	Malignant	11/23	47.8%	44.0%
	Mucinous adenocarcinoma		0/2	0.0%	
Breast	Invasive ductal carcinoma	Malignant	1/23	4.3%	4.2%
	Mixed lobular and duct carcinoma		0/1	0.0%	
Liver	Hepatocellular carcinoma	Malignant	1/40	2.5%	2.5%
Lung	Adenocarcinoma	Malignant	3/15	20%	20%
	Squamous cell carcinoma		2/10	20%	
Lymph node	Metastatic adenocarcinoma	Metastasis	13/40	32.5%	32.5%
Ovary	Serous adenocarcinoma	Malignant	0/33	0.0%	5.0%
	Mucinous adenocarcinoma		2/6	33.3%	
	Endometrial adenocarcinoma		0/1	0.0%	
Pancreas	Duct adenocarcinoma	Malignant	11/24	45.8%	48.0%
	Papillary adenocarcinoma		1/1	100.0%	
Prostate	Adenocarcinoma	Malignant	1/25	4.0%	4.0%
Uterus	Endometrial adenocarcinoma	Malignant	10/40	25.0%	25.0%
Others	Squamous cell carcinoma	Malignant	1/32	3.1%	3.1%

**Table 5:** Anti-HFG mAb epitope expression in human cancer cell lines

Type	Cell line	anti-HFG mAb binding
Gastric cancer	AGS	+ + +
	TSGH9201	+
	NCI-N87	+ +
	KATO III	−
	MKN45	−
	MKN74	−
	MKN7	−
Colon cancer	LOVO	−
	COLO 205	−
	COLO 201	+
	SW1116	+ + +
	DLD-1	+ +
	LS 174 T	+
	HT-29	−
	T84	−
Breast cancer	MCF-7	+ +
	MDA-MB-231	−
	MDA-MB-453	−
	T-47D	−
Ovarian cancer	NIH: OVCAR-3	+
	SW626	+
	SK-OV-3	−
	ES-2	−
Pancreatic cancer	SU.86.86	−
	EBC-1	−
	PANC-1	−
Lung cancer	NCl-H146	+
	NCl-H209	−
Skin cancer	A431	−

### CRC tumor growth is inhibited by anti-HFG mAb in a xenograft model

**Fig. 4:**
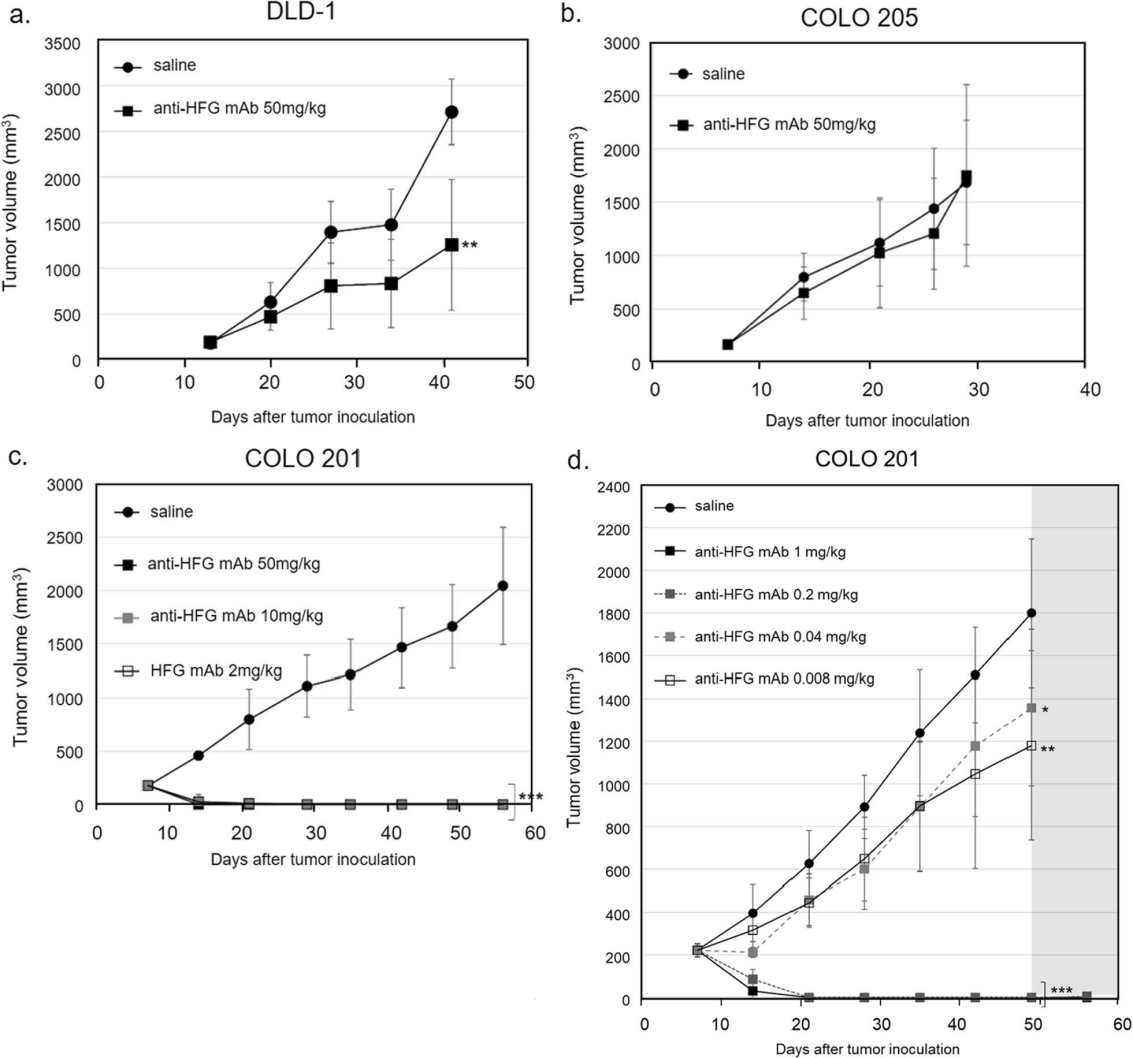
Inhibition of tumor cell proliferation by anti-HFG mAb in a xenograft model. Use of anti-HFG mAb effectively inhibits growth of DLD-1 cells **a** but not proliferation of COLO 205 cells (**b**). Dose-dependent inhibitory effects of anti-HFG mAb are demonstrated in COLO 201 cells (**c**, **d**)

The tumor-bearing SCID mice were given intraperitoneal anti-HFG mAb injections at doses from 2 to 50 mg/kg twice weekly for 6 weeks. Xenograft experiments were conducted to assess DLD-1, COLO 205, and COLO 201 tumor growth. The results indicated that anti-HFG mAb effectively reduced the growth rate of DLD-1 cells relative to the control (Fig. 4a) but did not inhibit growth of the COLO 205 cells (Fig. 4b); these findings are consistent with the binding ability of anti-HFG mAb (Table 5). Furthermore, a dose-dependent effect of anti-HFG mAb on COLO 201 cell growth was observed. The results obtained from the tumor-bearing SCID mice given intraperitoneal injections of anti-HFG mAb at doses from 2 to 50 mg/kg twice weekly for 6 weeks revealed significant tumor growth inhibition relative to the control group. Tumor volumes shrank rapidly and had completely remitted 2 weeks after tumor inoculation (Fig. 4c). We further reduced the treatment dose to 0 to 1-mg/kg anti-HFG mAb. The inhibitory effects were favorable when the concentration was > 0.2 mg/kg. Collectively, the results indicated that the antibody-based treatments specifically targeted fucosylated antigens to inhibit tumor growth. Thus, antifucosylation is a crucial mechanism, and developing an effective agent to target fucosylated antigens is a promising method for treating CRC.

### Combined chemotherapy and anti-HFG mAb cause synergistic cytotoxicity in CRC

**Fig. 5:**
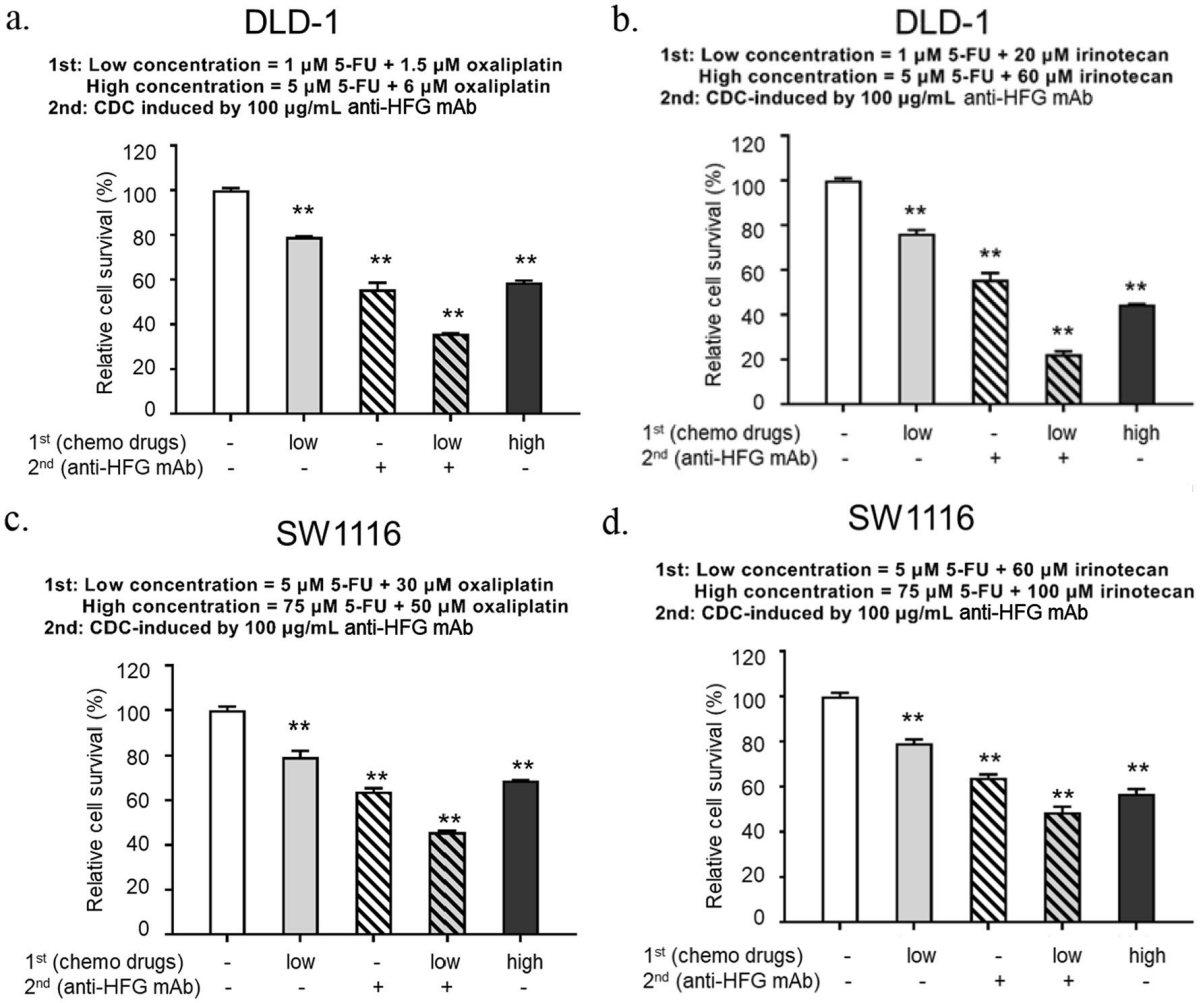
In vitro cytotoxic assays of chemotherapy drugs combined with anti-HFG-induced CDC in DLD-1 and SW1116 cells. DLD-1 **a**, **b** and SW1116 **c**, **d** cells are treated with either vehicle, 5-FU plus oxaliplatin/irinotecan, or the combination of 5-FU plus oxaliplatin/irinotecan for 48 h followed by CDC induced by 100-μg/mL anti-HFG mAb for 4 h. Data are expressed as mean ± standard deviation of duplicates. *CDC* complement dependent cytotoxicity

The combinatory effect of chemotherapy drugs with the CDC activity of anti-HFG mAb was induced in DLD-1 and SW1116 human colorectal adenocarcinoma cell lines that expressed HFGs recognized by anti-HFG mAb. We found that 100 μg/mL anti-HFG mAb applied to DLD-1 cells resulted in approximately 40% inhibition of cell growth (Fig. 5a). The maximal cell-killing effect was produced when the DLD-1 cells were first treated with a low concentration of 5-FU plus oxaliplatin followed by anti-HFG mAb-induced CDC. In addition, the combination treatment exhibited higher killing capacity than the treatment of only a high concentration of 5-FU plus oxaliplatin. Similarly, a low concentration of 5-FU with irinotecan combined with anti-HFG mAb-induced CDC also produced the maximal cytotoxicity (Fig. 5b). The results of the experiments involving SW1116 cells were consistent with those of the experiments involving DLD-1 cells (Fig. 5c, d). SW1116 cells treated with a low concentration of 5-FU plus oxaliplatin or a low concentration of 5-FU plus irinotecan followed by anti-HFG mAb-induced CDC exhibited the maximal cell growth inhibition. The cytotoxic assay results suggested that the combination of chemotherapy drugs with anti-HFG mAb resulted in the maximal cytotoxicity in DLD-1 and SW1116 cells relative to the use of either chemotherapy drugs or anti-HFG mAb alone. At present, these chemotherapy drugs are used clinically to treat CRC, and anti-HFG mAb can recognize the HFGs expressed in CRC. Therefore, anti-HFG mAb can be a potent treatment adjunct to these chemotherapy agents in future combination therapy regimens.

## Discussion

Lewis antigens are fucose-containing carbohydrates that are present in blood cells and normal epithelial cells, and they have been reported to be overexpressed on the surface of cancer cells [16]. The levels of Lewis antigens associated with tumor-related fucosylated epitopes (e.g., Lewis y, Lewis x, and sialyl Lewis^X^) are increased in various cancers (e.g., gastrointestinal cancers), and several Lewis antigens are related to survival and metastasis [17]. In the present study, HFG, a new fucosylated epitope, was assessed in an initial examination of 30 Taiwanese patients with CRC. Our results indicate significantly higher expression of HFGs in CRC tumor tissues than in normal tissues. The IHC staining and protein level analysis results also consistently indicate a high level of HFG expression in CRC tumor tissues and a low level of HFG expression in NATs. HFG appears to be a potential marker of CRC. Additional in vitro and/or in vivo investigations are required to clarify the treatment effectiveness and adverse effects of HFG targeting. Importantly, this research proposes a novel therapeutic target, and initially proves that it is feasible and convincing.

FUTs are key regulators of glycosylation and essential for the terminal fucosylation of glycans, including Lewis antigens [18, 19]. One review summarized a series of studies that examined the correlations of fucosylation with tumor initiation, distant metastases, and disease progression; the review suggested that overexpression of fucosylated epitopes strengthens FUT expression in various cancers. Alterations of FUTs are also associated with the formation of various tumor antigens, with this formation promoting the EMT properties of tumor cells and, thus, contributing to hematogenous metastasis [20]. An early study also demonstrated that the mRNA expression of FUT1 and FUT4 is increased in CRC tissues [21]. In the present study, HFG antigens were discovered in most CRC tumor tissues but were only detected in 30% of NATs. The glycans recognized by the anti-HFG mAb as requiring multiple fucoses in their structure appeared less frequently in normal tissues than in tumor tissues, and the altered expression of FUT is a possible reason for this phenomenon. Therefore, further explorations of FUTs and HFGs in CRC are warranted.

Various reports have revealed that aberrant glycosylation is involved in multiple human diseases, including cancer [22]. The results of our in vitro CDC experiments indicate that antibody therapies can inhibit tumor growth. Complement-dependent cytotoxicity is considered as a forceful defense mechanism of innate immunity, and activation of the classical complement cascade is triggered by the binding of C1q to the Fc region of a cell-bound antibody [23]. In our animal experiments, although DLD-1 cell line had a stronger binding ability to anti-HFG than COLO 201 in IHC staining, anti-HFG mAb exhibited more prominent cytotoxic effects in COLO 201 compared with those in DLD-1. The disparity might be due to the inconsistency of binding affinity between antibodies and complements. It was reported that the kinetic pathway of IgG oligomerization and complement activation might remarkably be altered by the cell surface antigen density and membrane mobility [24]. In addition, low affinity of Fc to C1q and the structural intricacy of the IgG1-C1q complex primarily cause the enhancement of antibody-mediated complement activation challenging [25].

Systemic therapies (e.g., chemotherapy, targeted therapy, and immunotherapy) are clinically used to relieve symptoms and prolong the lives of patients with mCRC. The use of targeted agents against tumor angiogenesis and EGFR is approved by the European Medicines Agency and US Food and Drug Administration (FDA) for the first- or second-line treatment of mCRC [26]. However, rat sarcoma virus (RAS) and B-Raf proto-oncogene serine/threonine kinase (BRAF) gene mutations, which occur in approximately 50% and 10% of mCRC cases, respectively, are predictors of resistance to EGFR inhibitors [27–29]. Thus, anti-EGFR therapy is only allowed for patients with mCRC with wild-type RAS and BRAF genes [3, 30, 31]. However, other mechanisms of resistance to anti-EGFR drugs have been observed in these patients [32, 33]. Angiogenesis is a key modulator involved in tumor cell proliferation, migration, and invasion; therefore, the antiangiogenesis strategy can also be applied as a targeted therapy for mCRC [34]. Because VEGF plays a crucial role in cancer angiogenesis [35], the FDA approved bevacizumab as the first anti-VEGF agent for mCRC in 2004 [36]. Anti-VEGF drugs are used in first or subsequent lines of mCRC treatment, and they are recommended for patients with mCRC with mutant RAS or BRAF genes [37]. However, several adverse effects contribute to VEGR blockades; they include hypertension, bleeding, and arterial thromboembolic events [38]. Immunotherapy is another emerging therapeutic option for mCRC, but it is only beneficial for tumors with mismatch repair deficiency or high microsatellite instability, which only comprise approximately 5% of mCRC cases [39, 40]. In fact, patients suitable for anti-EGFR, anti-VEGF, or immunotherapy only comprise of small portion of patients with CRC, whereas our results indicate significantly higher expression of HFG in most CRC tumor tissue than in normal tissue. These findings suggest a greater percentage of patients will be eligible for this novel treatment. Moreover, the low frequency of HFG expression in normal tissue suggests anti-HFG mAb may produce few side effects.

The antigen presentation of cancer cells in HFG is diverse, and the physiological significance of HFG is still unclear. Some studies have pointed out that colon-like cell lines like COLO 205, COLO 201, LS 174 T, and SW1116 might express Lewis-like antigen, and also upregulate the gene encoding glycosyltransferase, while undifferentiated cell line such as DLD-1 is less likely to express fucosylated antigens [41, 42]. Another highly glycosylated protein, mucin, represents the major secreted substance of the gastrointestinal tract, and major secreted product of colorectal cancer cells. Mucin family contains 22 characterized glycosylated macromolecules in human [43]. Some studies have also pointed out that patients with mucinous colorectal cancer have poorer prognosis, higher invasiveness and metastatic ability [44]. Although genetic alterations and tumorigenesis processes remain unclear, mucinous differentiation of colorectal cancers are associated with high frequency of mutations in KRAS or BRAF [45]. Among mucin protein family, MUC1 was reported to exhibit a role in tumorigenesis by cell death inhibition and metastasis promotion [46, 47]. One study combined monoclonal antibodies against MUC1 with chemotherapeutic agents and showed combined therapy applied in DLD-1 cells induced more apoptosis compared with monotherapy [48]. This report is partly consistent with the current IHC staining result in DLD-1 cells. Although detailed causes of HFG expression and mucinous differentiation in colorectal cancer cells remain indistinct, the current study reveals a novel treatment strategy.

We assessed the cytotoxicity of anti-HFG mAb and revealed the synergistic cytotoxic effects of anti-HFG mAb combined with chemotherapy. Another well-known mechanism of antibody-related drugs, namely antibody-dependent cellular cytotoxicity [49], should also be explored in future studies to assess the cytotoxicity of anti-HFG mAb in combination with chemotherapy or other FDA-approved drugs. We performed only in vitro assays in the present study; however, pharmacokinetics, pharmacodynamics, immunological interactions of antibodies, and other factors in a tumor microenvironment can influence the effects of therapeutic drugs [50]. Thus, additional ex vivo or in vivo investigations are required to clarify the therapeutic effectiveness of HFG targeting. For extensive applications involving the targeting of HFG antigens in cancer, antibody–drug conjugates and bispecific antibodies can also be developed to improve the effectiveness of treatments [51, 52]. The high tumor-specific antigen, HFG, can also be used as a cancer target in chimeric antigen receptor T-cell therapy [53].

It is difficult to evaluate the correlation between the expression levels of HFG and clinical features due to the small sample size. However, a preliminary analysis of our data reveals no correlation of IHC intensity with cancer stage, cancer type, differentiation of tumor, or CEA levels. Addressing the heterogeneity of HFG expression in cancer tissues and identifying possible biomarkers are crucial for the translation of HFGs' use as markers or targets into clinical practice. One possible approach is to analyze the gene expression of FUTs, which play a significant role in HFG biosynthesis, in tumor tissues. By identifying the major regulator of HFG overexpression, it becomes possible to use it as a biomarker for selecting an appropriate subgroup of patients who are more likely to benefit from HFG-targeted therapy. Moreover, in addition to IHC staining of tumor tissues to evaluate individuals with high HFG expression, assessing FUT gene expression can help further refine the patient population for targeted HFG therapy. Overall, these additional studies and subgroup identification efforts are crucial to provide evidence supporting the clinical utility and applicability of the current findings, facilitating the development of new targeted therapies, and extending the use of HFGs in cancer treatments.

In conclusion, we revealed that HFG is a cancer-associated antigen that is overexpressed in CRC tumor tissues. Our results demonstrated the CDC activity of anti-HFG mAb against CRC cell lines and revealed the higher cytotoxicity that is achieved when anti-HFG mAb is combined with clinical chemotherapy regimens. In clinical situation, if it is confirmed by pathological examination and IHC staining from the patient's tumor tissue that the tumor has high expression of HFG, it will be suitable for anti-HFG treatment. Furthermore, our findings serve as a basis for developing a new targeted therapy or extending the applicability of HFGs in other cancer therapies.

## Supplementary Information

**Additional file 1: Table S1.** Summary of colorectal cancer cell lines included in the study with mutational status. **Table S2.** Clinical and pathological parameters in the colorectal cancer patient cohort.

## Data Availability

The dataset supporting the conclusions of this article is included within the article.
