# Supplementary material for: Novel heavily fucosylated glycans as a promising therapeutic target in colorectal cancer
Source: J Transl Med. 2023 Jul 26;21:505. doi: 10.1186/s12967-023-04363-5 (PMC10373344; doi:10.1186/s12967-023-04363-5)
Supplement: Supplementary file 1 — Additional file 1: Table S1. Summary of colorectal cancer cell lines included in the study with mutational status. Table S2. Clinical and pathological parameters in the colorectal cancer patient cohort. [file 12967_2023_4363_MOESM1_ESM.docx]

**Additional file 1: Table S1.** Summary of colorectal cancer cell lines included in the study with mutational status

| **CRC cell line** | **Mutant genes** |
| --- | --- |
| LOVO | APC, FBXW7, KRAS, MSH2 |
| COLO 205 | APC, BRAF, SMAD4, TP53 |
| COLO 201 | APC, BRAF, SMAD4, TP53 |
| SW1116 | APC, KRAS, TP53 |
| DLD-1 | APC, KRAS, PIK3CA, TP53, |
| LS 174T | APC, KRAS, BRAF, PIK3CA |
| HT-29 | APC, BRAF, PIK3CA, SMAD4, TP53 |
| T84 | APC, KRAS, PIK3CA, TP53 |

**Additional file 1: Table S**2. Clinical and pathological parameters in the colorectal cancer patient cohort

| **Tissue** | | | | **NAT** | | **Tumor** | | **CEA ng/ml [<4.70]** | |  |  |  |
| --- | --- | --- | --- | --- | --- | --- | --- | --- | --- | --- | --- | --- |
| **No.** | **Age** | **Gender** | **Tumor site** | **Score** | **%** | **Score** | **%** | **Pre-OP** | **Post-OP** | **Pathology** | **Differentiation** | **Cancer stage** |
| 1 | 64 | M | Sigmoid | 0 | - | N/A | N/A | 3.58 | 1.94 | Adenocarcinoma | Moderately | IIIB |
| 2 | 67 | M | Rectum | 0 | - | 1 | 40% | 8.21 | 3.11 | Adenocarcinoma | Moderately | I |
| 3 | 75 | F | Rectal-sigmoid | 0 | - | 2 | 100% | 2.57 | 2.64 | Mucinous adenocarcinoma | Poorly differentiated | IIIB |
| 4 | 71 | M | Descending | 0 | - | 2 | 80% | 2.8 | 2.9 | Adenocarcinoma | Well-differentiated | IIA |
| 5 | 64 | F | Sigmoid | 0 | - | 2 | 80% | 3.58 | 1.94 | Adenocarcinoma | Moderately | IIIC |
| 6 | 54 | F | Rectum | 0 | - | 1 | 30% | 2.2 | 1.2 | Adenocarcinoma | Moderately | IIIB |
| 7 | 51 | F | Sigmoid | 0 | - | 2 | 30% | 3.58 | 1.94 | Adenocarcinoma | Moderately | IIA |
| 8 | 79 | M | Ascending | 3 | 80% | 3 | 100% | 2.2 | 1.2 | Adenocarcinoma | Moderately | I |
| 9 | 53 | M | Rectum | 0 | - | 1 | 60% | 8.21 | 3.11 | Adenocarcinoma | Moderately | IIIB |
| 10 | 43 | F | Cecum | 1 | 10% | 2 | 60% | 2.2 | 1.2 | Mucinous adenocarcinoma | Moderately | IVC |
| 11 | 76 | F | Ascending | 3 | 95% | 2 | 15% | 2.2 | 1.2 | Mucinous adenocarcinoma | Moderately | IIA |
| 12 | 63 | M | Sigmoid | 2 | 70% | 3 | 100% | 3.58 | 1.94 | Adenocarcinoma | Moderately | IIIB |
| 13 | 74 | M | Sigmoid | 0 | - | 0 | - | 3.58 | 1.94 | Adenocarcinoma | Moderately | IIA |
| 14 | 70 | M | Descending | 0 | - | 2 | 100% | 3.58 | 1.94 | Adenocarcinoma | Moderately | IIB |
| 15 | 76 | M | Sigmoid | 1 | 15% | 1 | 100% | 3.58 | 1.94 | Adenocarcinoma | Moderately | IIIB |
| 16 | 52 | F | Cecum | 3 | 60% | 2 | 100% | 2.2 | 1.2 | Adenocarcinoma with focal mucin production | Moderately | IIA |
| 17 | 69 | M | Transverse | 0 | - | 1 | 60% | 7.39 | 2.6 | Adenocarcinoma | Poorly differentiated | IIIB |
| 18 | 58 | M | Rectal-sigmoid | 0 | - | 2 | 90% | 2.2 | 1.2 | Adenocarcinoma | Moderately | IIA |
| 19 | 43 | F | Ascending | 3 | 70% | 3 | 100% | 2.2 | 1.2 | Adenocarcinoma | Moderately | IIA |
| 20 | 83 | F | Sigmoid | 0 | - | 1 | 10% | 3.58 | 1.94 | Adenocarcinoma | Moderately | IIA |
| 21 | 79 | M | Sigmoid | 0 | - | 0 | - | 3.58 | 1.94 | Adenocarcinoma | Moderately | I |
| 22 | 65 | F | Sigmoid | 0 | - | 1 | 20% | 3.58 | 1.94 | Adenocarcinoma | Moderately | IIA |
| 23 | 59 | M | Sigmoid | 0 | - | 1 | 80% | 3.58 | 1.94 | Adenocarcinoma | Moderately | IIIB |
| 24 | 53 | M | Ascending | 3 | 100% | 2 | 75% | 2.2 | 1.2 | Adenocarcinoma | Moderately | IVC |
| 25 | 71 | F | Ascending | 3 | 30% | 2 | 100% | 2.2 | 1.2 | Adenocarcinoma | Moderately | I |
| 26 | 64 | M | Sigmoid | 0 | - | 1 | 40% | 2.61 | 2.14 | Adenocarcinoma | Well-differentiated | I |
| 27 | 67 | F | Sigmoid | 0 | - | 2 | 100% | 3.58 | 1.94 | Adenocarcinoma | Moderately | IIA |
| 28 | 75 | M | Rectum | 0 | - | 0 | - | 8.21 | 3.11 | Adenocarcinoma with focal mucin production | Moderately | IIA |
| 29 | 71 | M | Sigmoid | 0 | - | 2 | 80% | 3.58 | 1.94 | Adenocarcinoma | Moderately | IIA |
| 30 | 40 | M | Sigmoid | 0 | - | 1 | 20% | 17.35 | 1.67 | Adenocarcinoma | Moderately | IVA |
